# Recent Advances, Bottlenecks, and Future Directions in *Plasmodium falciparum* Vaccine Development

**DOI:** 10.3390/vaccines14030277

**Published:** 2026-03-21

**Authors:** Gulbuse Turan, Maxence J. Boggio, Ahmad Syibli Othman, Victory Nnaemeka, Adrian V. S. Hill, Ahmed M. Salman

**Affiliations:** 1 Jenner Institute, University of Oxford, Oxford OX3 7DQ, UK; maxence.boggio@ndm.ox.ac.uk (M.J.B.); syibli.othman@ndm.ox.ac.uk (A.S.O.); victory.nnaemeka@ndm.ox.ac.uk (V.N.); adrian.hill@ndm.ox.ac.uk (A.V.S.H.); 2 School of Biomedical Sciences, Faculty of Health Sciences, Universiti Sultan Zainal Abidin, Kuala Nerus 21300, Malaysia

**Keywords:** global health, malaria, parasites, vaccines, virus-like particles

## Abstract

Malaria remains a major global health burden, with an estimated 282 million cases and 610,000 deaths reported in 2024, disproportionately affecting children under five years of age and pregnant women in sub-Saharan Africa. Although antimalarial drugs are highly effective at clearing infections, their reliance on timely diagnosis and treatment limits their scalability as a population-wide control strategy. Vaccines therefore represent a critical tool for reducing malaria-associated morbidity and mortality, as well as interrupting parasite transmission, by inducing durable protective immunity. However, the complex lifecycle of *Plasmodium* parasites poses significant challenges for vaccine development, including the identification of protective antigens and optimal vaccine formulations. In this review, we summarize current vaccine strategies and discuss their key limitations. We also highlight emerging opportunities for possible avenues for future research and development.

## 1. Introduction

Malaria is a life-threatening disease caused by *Plasmodium* parasites that are transmitted from person to person by the bites of infected *Anopheles* mosquitoes. There are five *Plasmodium* species that can cause malaria in humans; *P. falciparum*, *P. vivax*, *P. malariae*, *P. ovale* and zoonotic *P. knowlesi*. Two of these species, *P. falciparum* and *P. vivax*, are responsible for most cases of human mortality and morbidity [1]. The World Health Organization (WHO) reported that in 2024, there were an estimated 282 million cases of malaria in 83 countries, an increase of 19 million cases from 2023 [2]. Malaria deaths reached 610,000 in 2024. Despite decades of control efforts, the persistently high mortality underscores the urgent need for more effective and durable interventions, particularly vaccines that can interrupt infection at multiple stages of the parasite lifecycle.

**Figure 1:**
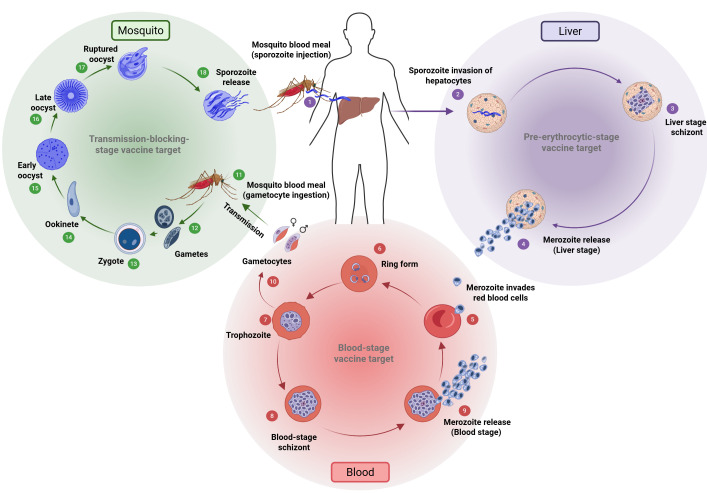
The lifecycle of *Plasmodium falciparum*. Sporozoites transmitted by an infected *Anopheles* mosquito (1) initiate liver-stage infection and development (2–4), followed by asexual blood-stage replication in erythrocytes (5–9). A subset of parasites differentiates into gametocytes (10), which are taken up by mosquitoes during a blood meal (11) and undergo sporogonic development (12–18), completing the transmission cycle. Figure was created via BioRender.com.

The lifecycle of *Plasmodium* can be broken down into three major stages: pre-erythrocytic, blood, and mosquito. Each stage of its development presents distinct biological mechanisms that not only allow the parasite to thrive but also serve as potential targets for intervention. Transmission occurs when an infected mosquito injects sporozoites into the human bloodstream, initiating the pre-erythrocytic stage. This stage is particularly crucial, as it represents the parasite’s first major replication phase in humans and a key opportunity for prophylactic intervention. After liver invasion, the parasite undergoes asexual replication followed by the blood stage, which is responsible for the clinical manifestations of malaria [3]. The cycle is completed when sexual forms (gametocytes) are taken up by a mosquito, continuing transmission. Figure 1 illustrates the complex lifecycle of *P. falciparum*.

Multi-stage vaccines that act at several points in the *Plasmodium* lifecycle are increasingly regarded as a leading strategy for achieving robust, long-lasting malaria protection while also reducing transmission. With the WHO recommendation and prequalification of two pre-erythrocytic malaria vaccines, the development of complementary vaccines targeting blood-stage and transmission-blocking stages has become crucial to enhance overall impact on disease and transmission.

## 2. Current Situation

Table 1 below shows the ongoing clinical trials against several antigens of *Plasmodium falciparum* that have been registered to the ClinicalTrials.gov database as of March 2026.

### 2.1. Pre-Erythrocytic Stage Vaccines

Vaccination efforts against malaria have increasingly focused on preventing infection at its earliest stages before the parasite reaches the bloodstream. Pre-erythrocytic malaria (PEM) vaccines are designed to intercept *Plasmodium* falciparum sporozoites in the skin or liver, targeting antigens specific to these stages to halt progression. Among these, RTS,S (GSK) is the first, built to display 18 NANP repeats and the carboxyl terminus of CSP on Hepatitis B surface antigens alongside HepB-S protein [4,5]. It is formulated with the AS01 liposome-based adjuvant system, which contains monophosphoryl lipid A (MPLA) and QS-21 (*Quillaja saponaria*-fraction 21), to enhance immunogenicity by stimulating strong humoral and cellular responses [4]. During Phase III trials of RTS,S, it was administered to children aged 5 to 17 months on a schedule of 0, 1, 2, and 20 months, resulting in about a 36% reduction in clinical malaria over the 48 months following the first dose [6,7]. Although this efficacy was modest, the magnitude of the malaria burden was such that a vaccine demonstrating 26–36% protection was considered sufficient to warrant WHO recommendation and subsequent implementation in multiple African countries [7]. In high-transmission settings, even partial protection can result in meaningful reductions in clinical incidence, severe disease, and malaria-associated mortality at the population level. In 2021, WHO recommended the use of RTS,S/AS01 (Mosquirix^TM^, GSK, Singapore), which led to a 13% decrease in overall childhood mortality, even with less than 50% coverage of the four-dose regimen [8]. However, RTS,S has key limitations, which include rapid waning immunity and misalignment between peak antibody responses and malaria transmission seasons [9]. The partial protection conferred by RTS,S does not completely prevent new infections or reinfections in endemic settings [10]. Alongside clinical trials, quantitative modeling has also demonstrated the biphasic waning of anti-CSP antibody titers, with declining titers predicting reduced efficacy and more breakthrough infections [11]. To address the shortcomings of RTS,S, alternative strategies have been explored, including the seasonal administration of RTS,S, which aims to synchronize vaccine-induced protection with transmission peaks [12]. Clinical studies have demonstrated that the RTS,S malaria vaccine primarily induces modest and short-lived CD4^+^ T cell responses, characterized by the production of IL-2, TNF-α, and (to a lesser extent) IFN-γ [13]. These T cell responses are directed mainly against the C-term of CSP. Several studies have measured increases in the frequencies of CSP-specific CD4^+^ T cells producing IL-2 and TNF-α after vaccination; central memory and effector CD4^+^ T cell pools are expanded, but the responses diminish over time and are not considered very robust or long-lasting [14]. On the economical side, pilot projects in Ghana, Kenya and Malawi indicate that national immunization programs can deliver the RTS,S vaccine for just USD 2.3–3.0 per dose, and the all-in economic cost of USD 8–10 per dose is comparable to what countries already pay for other routine childhood vaccines [15].

On the other hand, preclinical studies of R21 (University of Oxford and Serum Institute of India) showed its effectiveness, with sterile protection against transgenic *P. berghei* parasites containing PfCSP in BALB/c mice with three vaccine doses [16]. CHMI studies in adults have shown that vaccination followed by challenge leads to an expansion of circulating T follicular helper (cTfh) cells (CXCR5^+^/PD-1^+^). This expansion predominantly involves Th2/Th17-polarized subsets, which are linked to strong antibody production and the activation of naïve B cells [17]. In 2023, R21 was first approved in Ghana, Nigeria, and Burkina Faso; then, the WHO subsequently recommended its use for the same target populations and immunization schedules as RTS,S. Phase III trials showed that R21, formulated with Matrix-M (Serum Institute of India, Pvt. Ltd. (Pune, India); Novavax, Inc. (Gaithersburg, MD, USA)), was safe for children aged 5 to 36 months when given on a 0, 1, and 2-month schedule [18]. Vaccine efficacy over 24 months of R21 was reported as 75% and 77% for the time to the first episode and for multiple episodes of clinical malaria, respectively [19,20]. CD4^+^ T cells secrete cytokines such as IL-2, TNF-α, and IFN-γ; however, the overall magnitude of these responses is modest and typically short-lived. Their primary role is the facilitation of antibody production rather than serving as direct effectors of protection [21]. In terms of cost-effectiveness, with a ceiling price of USD 3 per dose, the R21/Matrix-M vaccine prevents a disability-adjusted life-year (DALY) for USD 30–34, ranking it among the most economically efficient health measures in malaria-endemic Africa [22].

One of the attenuated whole sporozoite vaccines, radiation-attenuated PfSPZ by Sanaria has demonstrated high efficacy in the CHMI study [23]. However, as per other PEM vaccines, PfSPZ too did not stimulate durable antibody response over time [24]. Genetically attenuated sporozoite vaccine, PfSPZ-GA1, did not provide optimal efficacy; yet the antibody response it provided was higher than radiation-attenuated PfSPZ [25]. The labor-intensive process required to extract sporozoites, combined with the high manufacturing costs of whole sporozoite vaccines, presents significant challenges for large-scale production. Moreover, the need for intravenous (IV) administration further complicates deployment, particularly in resource-limited settings such as many regions of sub-Saharan Africa. While whole sporozoite vaccines may hold promise as a travel vaccine for non-endemic populations, their practicality for widespread use in endemic areas remains limited due to these logistical and economic constraints.

Meanwhile, viral vector-based approaches seek to enhance T cell responses by combining them with VLP vaccines to increase their efficacy [26].

Recent efforts in structural biology are shaping a more refined understanding of protective epitopes within CSP, offering a path to more targeted and effective vaccine design. Still, antigenic diversity within CSP and the complex immune evasion tactics of the parasite continue to limit the breadth and longevity of vaccine-induced protection. Overcoming these hurdles will be essential for the development of next-generation PEM vaccines with broader coverage and more durable efficacy.

### 2.2. Blood-Stage Vaccines

Current blood-stage malaria vaccine development primarily focuses on generating antibodies that can block the merozoite invasion of RBCs [27]. Recent breakthroughs have revitalized this field, most notably with the RH5.1/Matrix-M vaccine, the first blood stage candidate to demonstrate significant clinical efficacy in endemic populations [27,28]. RH5.1 is a soluble protein vaccine that targets the highly conserved *P. falciparum* reticulocyte-binding protein homolog 5 (PfRH5). Phase Ib trials in Burkina Faso have shown that the vaccine has a favorable safety and immunogenicity profile in both adults and young children living in malaria-endemic regions [28]. Another Phase I/IIa trial using recombinant PfRH5 with the AS01B adjuvant revealed not only strong immune responses but also durable antibody levels sustained for over two years, along with a measurable reduction in parasite growth during CHMI [29]. Vaccination strategies utilizing heterologous viral vectors (ChAd63/MVA-RH5) have achieved antibody titers in humans comparable to or exceeding those found in individuals with long-term natural exposure [30]. A modified version of RH5.1, RH5.2 has now been produced as a VLP vaccine candidate and has entered clinical trials [31,32].

In contrast, other blood-stage targets like MSP1, MSP3, AMA1, and EBA-175, despite their critical roles in erythrocyte invasion, are highly polymorphic [33,34]. This variability has limited their effectiveness as vaccine antigens, with clinical studies showing either minimal or no protection despite high antibody titers [35,36,37]. In parallel, researchers have explored a whole-parasite blood-stage vaccine, including lysates of parasitized RBCs [38]. The study showed that immunization with a lysate derived from P. yoelii blood-stage parasites significantly reduced parasitemia and increased survival in mice as well as reducing the parasite loads in the liver after blood-stage challenge [38].

### 2.3. Transmission-Blocking Vaccines

Transmission-blocking vaccines (TBVs) target the stages of parasite development that occur within the mosquito rather than directly preventing disease in humans. TBVs work by inducing antibodies against antigens expressed during the parasite’s sexual stage, thereby disrupting the transmission cycle [39]. Among the most extensively studied candidates is Pfs25, a protein expressed on the surface of zygotes and ookinetes. Although preclinical studies demonstrated strong immunogenicity, clinical trials using Pfs25 in various formulations, such as recombinant protein, fusion protein, and virus-like particles, have yielded only modest success [40,41]. Enhancing Pfs25 immunogenicity has been attempted through fusion with the molecular adjuvant IMX-313, which significantly improved both antibody responses and transmission-blocking activity in comparison to its monomeric form [41]. Furthermore, combining Pfs25 with the pre-erythrocytic vaccine RTS,S in a multistage vaccine approach was proven to be safe, showing no interference in the functional antibody responses against either antigen in vitro [40,41]. Beyond Pfs25, several other TBV candidates are under development, including Pfs230 as well as Pfs48/45 (in Phase I/II trials) [42]. These antigens offer additional avenues to interrupt the parasite life-cycle by blocking parasite development within the mosquito vector. For example, TBV candidates such as Pfs25 or Pfs230 target parasite stages expressed in the mosquito midgut and induce antibodies that prevent fertilization or ookinete development after a mosquito blood meal. When a mosquito feeds on an immunized individual, these antibodies can inhibit parasite maturation in the mosquito, thereby preventing onward transmission to new hosts. When combined with pre-erythrocytic or blood-stage vaccines that protect individuals from infection or disease, such trans-mission blocking strategies could help reduce parasite circulation in the population and contribute to a more comprehensive malaria elimination strategy.

### 2.4. Placental Malaria Vaccines

Placental malaria results from the sequestration of *Plasmodium falciparum*-infected erythrocytes in the intervillous space of the placenta by binding to chondroitin sulphate A (CSA). This process leads to maternal anemia, low birth weight, still birth, and fetal growth restriction [43]. Up to 40% of pregnant women in endemic regions may develop placental malaria, which may cause substantial maternal and infant mortality [44]. Women acquire protection over successive pregnancies through the gradual development of antibodies targeting the parasite protein VAR2CSA, which mediates CSA binding [43]. Given the parity-dependent nature of immunity, effective placental malaria vaccination must be administered prior to the first pregnancy.

Two VAR2CSA-based candidates, namely PRIMVAC and PAMVAC, have completed Phase I clinical evaluation (NCT02658253 and NCT02647489, respectively). PRIMVAC, a recombinant protein vaccine derived from the 3D7 VAR2CSA sequence, was tested in malaria-naïve women in France and in nulligravid women in Burkina Faso [43]. The vaccine demonstrated an acceptable safety profile and induced seroconversion in all vaccinated participants. Antibodies recognized native VAR2CSA and the inhibited adhesion of homologous CSA-binding parasites; however, cross-reactivity against heterologous strains was limited and primarily observed at higher doses [43]. Similarly, PAMVAC, another recombinant protein vaccine based on the FCR3 variant VAR2CSA variant, showed favorable safety and induced functional adhesion-inhibitory antibodies in malaria-naïve adults [44]. As with PRIMVAC, the inhibitory activity of PAMVAC was largely strain-specific [44].

A major challenge in placental malaria vaccine development is the extensive polymorphism of VAR2CSA. Recent structure-guided design efforts have leveraged cryo-electron microscopy data to generate optimized immunogens retaining conserved CSA-binding elements while reducing structural complexity [45]. A redesigned construct demonstrated an improved production yield and induced potent adhesion-blocking antibodies comparable to full-length VAR2CSA in a pre-clinical study in rats [45]. Multivalent cocktail strategies incorporating variants from different parasite strains broadened functional inhibition, which might address a key limitation of earlier candidates [45]. Using epitope excision and quantitative mass spectrometry, Misal et al. identified conserved VAR2CSA epitopes preferentially recognized by multigravida women who had acquired functional anti-adhesion antibodies [46]. Protective IgG preferentially targets conserved epitopes near the CSA-binding site, many of which require a full-length VAR2CSA conformational structure for proper antibody recognition [46].

Future priorities include defining correlates of protection, improving cross-reactive functional responses, and assessing durability of vaccine induced immunity prior to first pregnancy. Advancing beyond allele-specific responses toward broadly inhibitory, conserved epitope targeting will be essential to achieve clinically meaningful protection against placental malaria.

## 3. Vaccine Platforms That Have Been Used in Malaria Research

Efforts to develop malaria vaccines have explored nearly all available platforms. Whole sporozoite vaccines have been tried as a first-generation vaccine platform, while recombinant proteins, subunit vaccines, and viral vectors have been tried as second-generation vaccines. Third-generation vaccines include nucleic acid vaccines and nanoparticle-based vaccines. VLPs, and a more recent platform, RNA-based vaccines, fall under the third generation [47]. All these platforms have been explored or are under development to target specific stages of the *Plasmodium* lifecycle independently/simultaneously, with each being tailored to disrupt the parasite at a distinct point.

Whole sporozoite vaccines target the parasite’s pre-erythrocytic stage, and they are produced by attenuating sporozoites through radiation, genetic modification, or chemical treatment [48]. Even though it has been shown that this platform provided sterile protection in humans, their reliance on complex production processes and delivery constraints, particularly the need for intravenous administration and their dose-dependent efficacy, limit their feasibility in large-scale immunization programs across endemic regions [47].

In contrast, viral vector-based platforms have not yet achieved consistent or sufficient protective efficacy in humans. Although heterologous prime-boost vaccination regimens have shown promise in murine studies [49] and demonstrated their ability to induce strong CD8^+^ T cell responses, overall protective efficacy has remained suboptimal in controlled human malaria infection (CHMI) studies [50,51,52].

VLP-based vaccines, on the other hand, have gained significant attention as nanoparticle-based platforms due to their excellent safety profile, ease of manufacturing, and suitability for widespread deployment [53]. Given recent advancements in structural biology, it is now possible to more accurately predict epitopes that specifically stimulate B and T cells to display within the particles [54]. The NANP repeat region of the circumsporozoite protein (CSP) antigen has been one of the primary targets in subunit malaria vaccine studies [55]. It continues to be evaluated today via all platforms possible, including both genetic fusion and chemical conjugation strategies for antigen display on VLP vaccine candidates.

While these breakthrough vaccines represent significant advancements, challenges remain, particularly regarding vaccine production, distribution, and affordability. Excessive costs associated with advanced vaccine technologies often leave vulnerable populations in low-income regions without access. In 2023, around 14.5 million children globally did not receive their first dose of the combined diphtheria/tetanus/pertussis (DTP) vaccine, commonly referred to as “zero-dose children” [56]. This metric is used by the WHO as a key indicator for monitoring global immunization gaps, highlighting that considerable progress is still required to achieve widespread access to vaccines. Global efforts such as the Global Alliance for Vaccines and Immunization (GAVI) and the Coalition for Epidemic Preparedness Innovations (CEPI) aim to address these disparities. The COVAX initiative, led by GAVI, WHO, and CEPI, aimed to provide equitable access to COVID-19 vaccines, delivering millions of doses to countries that lacked the resources to secure vaccines independently. Despite these efforts, continued investment and innovation are required to ensure that life-saving vaccines reach all populations, particularly in regions where vaccine-preventable diseases remain prevalent.

### 3.1. Attenuated Vaccines

Anattenuated malaria vaccine is based on *Plasmodium* parasites rendered non-pathogenic by radiation exposure, the co-administration of chemoprophylaxis, or targeted genetic alteration [57,58,59]. These strategies are designed to block the parasite’s lifecycle from advancing beyond the liver stage to the symptomatic blood stage, while simultaneously inducing strong pre-erythrocytic immune responses [60]. By mimicking natural infection, these vaccines induce strong humoral and cellular immune responses. Attenuated malaria vaccines remain a promising strategy for long-term protection, though further refinement of attenuation methods and delivery systems is needed to achieve broader effectiveness and practical application [61,62].

The most advanced whole-sporozoite vaccine candidate involves radiation-attenuated *P. falciparum* sporozoites, in which development is halted shortly after they invade hepatocytes. Despite their promising efficacy, these vaccines face several limitations. The production and storage of live attenuated *Plasmodium* parasites are technically demanding and require strict biosafety measures [63]. In addition, large numbers of sporozoites are required to achieve protection, which complicates large-scale manufacturing and limits feasibility for mass vaccination, particularly in resource-limited settings. Maintaining sporozoite viability during storage, transportation, and administration further adds logistical complexity. The need for intravenous administration also poses challenges for large-scale deployment [64]. Moreover, ensuring consistent attenuation without reversion to virulence remains a safety concern and immune responses induced by attenuated vaccines may vary among individuals and provide strain-specific rather than cross-species protection [65].

### 3.2. Recombinant Protein Vaccines

Recombinant protein malaria vaccines employ purified *P. falciparum* antigens to elicit immune responses without the use of whole organisms, thereby offering a favorable safety profile, including in immunocompromised individuals. Their defined composition, high purity and generally good physicochemical stability facilitate stringent quality control and large-scale manufacture [66]. Recombinant vaccines generally require robust physicochemical characterization and stability testing [67].

However, antigenic polymorphism and the limited durability of protection remain important challenges for blood and pre-erythrocytic stage candidates. Moreover, recombinant protein subunit vaccines typically exhibit lower intrinsic immunogenicity than whole-organism approaches and therefore require formulation with potent adjuvants and administration in multi-dose schedules to achieve robust and sustained antibody responses [68].

Among blood-stage vaccine candidates, RH5 has emerged as one of the most promising recombinant antigens. In a Phase I/IIa clinical study conducted in malaria-endemic populations, a ChAd63/MVA RH5 prime followed by RH5 protein boost demonstrated safety and induced high levels of growth inhibition activity (GIA), particularly in infants, representing some of the strongest functional antibody responses reported for a blood-stage candidate [69]. Another recombinant protein vaccine, FMP2.1/AS02(A), based on apical membrane antigen 1 (AMA1), induced robust antibody responses in Phase I and II trials; however, extensive antigenic polymorphism limited cross-strain efficacy (NCT00460525) [70]. Similarly, SumayaVac-1/GLA-SE, a full-length merozoite surface protein 1 (MSP1)-based formulation, demonstrated immunogenicity but modest protective outcomes (NCT05644067) [71]. The GMZ2 vaccine, a recombinant fusion protein combining glutamate-rich protein (GLURP) and merozoite surface protein 3 (MSP3), advanced to a multicenter Phase IIb trial in African children and showed good safety and immunogenicity but limited clinical efficacy [72].

Recombinant protein approaches have also been central to TBV development. Sexual-stage antigens such as Pfs25 and Pfs230 have been evaluated in early-phase clinical trials. Pfs25-based vaccines, which are also known as gamete vaccines, demonstrated safety and the induction of transmission-reducing antibodies in Phase I studies [73,74]; however, when compared head-to-head with Pfs230D1-EPA, a zygote vaccine formulated with Alhydrogel, it failed to induce durable serum functional activity [75].

### 3.3. Virus-like Particles (VLPs) as a Vaccine Platform

VLPs have gained renewed traction in malaria vaccine development due to their strong immunogenic profile, safety, and adaptability. Their structural similarity to viruses enhances immune recognition, and their ability to display multiple antigens may help when considering several antigens associated with the complexity of *Plasmodium*’s lifecycle. When combined with potent adjuvants, VLPs offer a versatile, scalable, and effective platform. VLPs are non-infectious, cage-like structures that mimic the morphology of viruses but lack any viral genetic material [53]. As such, they are incapable of replication or causing disease, making them a safe and effective platform for vaccine development [76]. Their ability to be genetically or chemically engineered to display antigens of interest on their surface has positioned VLPs as a versatile tool in vaccinology, especially for diseases where antibody-mediated immunity is essential for protection [77].

VLPs self-assemble once high levels of viral scaffold proteins, or chimeric fusions carrying foreign epitopes, are expressed in a host cell [78]. Their symmetry usually mirrors that of the original virus, and they display flexibility in assembly [79]. Although capsid/envelope proteins assemble into VLPs, core proteins can do the same, as seen in HBV where surface and core proteins form HBsAg and HBcAg VLPs, respectively. Certain VLPs even undergo reversible assemble/dissemble process, which makes them useful carriers for ssRNA, dsRNA, or polymers like polyglutamate [80]. Another key advantage of VLPs is their optimal size, typically 20–200 nm, which allows for efficient free drainage into the lymphatic system and access to draining lymph nodes (dLNs), where immune responses are orchestrated [78]. This size range also facilitates recognition and uptake by antigen-presenting cells (APC), such as dendritic cells (DC) [81]. Upon administration, VLPs are rapidly transported to lymph nodes, where they are internalized by APCs, processed, and presented via MHC molecules to activate T cells [82].

In parallel, unprocessed VLPs that reach B cell zones can cross-link B cell receptors, leading to the endocytosis, activation, and proliferation of B cells [83]. In conjunction with T helper cell support, this triggers immunoglobulin class switching and the development of long-lived memory B cells. Additionally, exogenous antigens can also be presented via the MHC class I pathway via cross-presentation [84]. By effectively mimicking natural infections without the risk of pathogenicity, VLPs can elicit robust humoral and cellular immune responses.

VLPs can be generated in several expression systems, each offering a distinct advantage among volumetric yield, manufacturing cost, and authenticity of post-translational modifications (PTMs). Yeast cells such as *Saccharomyces cerevisiae* or *Pichia pastoris* combine favorable economics with high-density fermentation and underpin the current industrial production of the RTS,S and R21 VLPs. Insect cells support elevated recombinant-protein expression and provide eukaryotic PTMs; however, they entail greater production costs and carry a risk of baculoviral contamination. This system was used in the production of an human papillomavirus (HPV) vaccine—Cervarix [85]. Mammalian cell lines like HEK293 and CHO provide human-like PTMs, albeit at higher cost with low yield. Plant cells (e.g., *Nicotiana benthamiana*) offer scalable and cost-efficient biomass but typically yield lower VLP expression [86]. Nevertheless, they have been used for COVID-19 and influenza vaccine research [87,88]. The bacteria system (e.g., *E. coli*) has been characterized as genetically tractable, rapidly expandable, and highly scalable, yet the resulting VLPs lack complex PTMs and may exhibit diminished immunogenicity [89]. Finally, an emerging cell-free expression system enables high throughput without contamination; however, current reagent costs and limited glycosylation fidelity constrain large-scale adoption [90]. It still might be promising, especially for on-demand personalized/cancer vaccines.

Two vaccines, RTS,S/AS01 and R21/Matrix-M, have been approved and are being implemented in several African countries. These vaccines share a common antigenic target, the central NANP repeat region and C-terminal region of the CSP, which is displayed on a VLP platform based on the hepatitis B virus surface antigen (HBsAg) [5,16]. Despite their similarities, there are two key differences between RTS,S and R21 that distinguish their design and immunogenic potential. Firstly, the density of CSP antigens displayed on the VLP surface differs substantially. RTS,S is composed of a mixture of CSP-HBsAg fusion proteins (the RTS component) and excess unfused HBsAg (additional S) in a 1:4 ratio [5]. This formulation results in VLPs with a relatively low density of CSP, as only 20% of the monomers display the antigen. In contrast, R21 is composed exclusively of CSP-HBsAg fusion proteins, meaning that 100% of the VLP subunits carry CSP, leading to a four-fold higher CSP density on the particle surface compared to RTS,S [16]. Secondly, the adjuvant systems used in each vaccine differ. RTS,S is formulated with AS01, a liposome-based adjuvant system that contains MPLA and QS-21, both of which activate innate immunity and enhance T cell and antibody responses [91]. R21, on the other hand, is formulated with Matrix-M, nanoparticle adjuvant composed of saponin fractions also derived from *Quillaja saponaria*, which has shown a strong ability to enhance both humoral and cellular immune responses while being well tolerated [20].

Regarding the best route of administration for VLP vaccines, intradermal delivery, particularly via microneedle or direct skin injection, is considered optimal due to superior immune activation, lymphatic targeting, and dose-sparing potential compared to intramuscular or subcutaneous routes. Recently, it was reported that the R21/Matrix-M subcutaneous route of administration R21/Matrix-M with immune complex elicits a robust immune response and demonstrates superior efficacy compared to the intramuscular route in a mouse model [92].

Several VLP-based vaccines have already been approved and are in use against human infectious diseases, including HPV [93,94], hepatitis B virus (HBV) [95], hepatitis E virus (HEV) [96], chikungunya [97] and malaria [9,18]. They also have even been successfully used in multivalent formulations, as demonstrated in the Gardasil9 vaccine [94]. Aside vaccine platform, VLPs have been explored for the targeted delivery of therapeutic agents, such as drugs and nucleic acids [98,99,100].

### 3.4. Viral Vectored Vaccines

A broad range of viral systems, including SV40, adenoviruses, poxviruses, lentiviruses, and herpesviruses, have been explored as vectors for antigen delivery. These platforms are typically engineered to be replication-deficient, ensuring safety while retaining their ability to express target antigens efficiently. Viral vectors can also be modified to target-specific cells or tissues, and their inherently immunogenic structural components often obviate the need for additional adjuvants. One of the most widely distributed viral-vectored vaccines to date is ChAdOx1 nCoV-19, developed against SARS-CoV-2, which demonstrated the feasibility of large-scale vector-based immunization [101].

In the malaria field, viral-vectored platforms, specifically simian adenovirus (ChAd63) and modified vaccinia Ankara (MVA), were originally advanced as stand-alone malaria vaccines to drive potent T cell responses against pre-erythrocytic and blood-stage antigens. Heterologous ChAd63/MVA regimens, expressing multi-epitope string-fused thrombospondin-related adhesion protein (ME-TRAP) or CSP,induced robust T cell responses and achieved partial protection in CHMI studies, demonstrating the strong cellular immunogenicity of these vectors even when sterile protection was not achieved [102]. The ChAd63/MVA-RH5 vaccine has also shown promising results, being well tolerated and highly immunogenic, with high vaccine-induced GIA reported in infants [69].

In recent years, the focus has partially shifted from vector-only regimens toward their use in combination strategies aimed at broadening protection. Pairing viral vectors with protein-in-adjuvant vaccines aims to marry strong vector-driven T cell responses with high-titer, functional antibodies [103]. However, when this approach was tested by co-administering ChAd63/MVA ME-TRAP with one of the leading VLP vaccines (R21/Matrix-M) in malaria-naïve UK adults, the combination regimen did not yield any additional efficacy beyond that achieved with R21/Matrix-M alone [21].

### 3.5. Nucleic Acid-Based Vaccines

As a highly adaptable and rapidly advancing platform, nucleic acid-based vaccines rely on the delivery of genetic material, either DNA or messenger RNA (mRNA), encoding key *Plasmodium falciparum* antigens, which are subsequently expressed in host cells to elicit immune response. As with other vaccine platforms, nucleic acid-based vaccines are also turning their attention to multivalent/multistage studies.

DNA vaccines were among the earliest nucleic acid-based strategies investigated against malaria. Several *P. falciparum* antigens, mainly CSP and Pfs25 or liver-stage antigen-1 (LSA-1) have been studied. Preclinical studies in murine models demonstrated the induction of antigen-specific T cell responses and moderate antibody titers. Reeder et al. have shown that ΔGPI (a synthetic DNA that was synthesized and then inserted into a pVax backbone with an added IgE leader sequence encoding CSP (3D7) without a GPI anchor) provided elevated levels of inhibition (84.6%) of liver infection in a mouse model [104]. However, early clinical trials in humans revealed limited immunogenicity. A first-in-human PfCSP DNA malaria vaccine clinical trial failed to trigger an antigen-specific antibody immune response [105]. A study tested a pentavalent DNA vaccine, encoding PfCSP, PfTRAP/SSP2, Pf-exported protein-1 (PfExp1), N′ and C′ termini of Pf liver stage antigen-1 (PfLSA1), and Pf liver stage antigen-3 (PfLSA3) combined with human granulocyte macrophage-colony stimulating factor (hGM-CSF) to improve immunogenicity, and found that the DNA vaccine was safe but not protective against malaria infection [106]. When boosted with viral vectored vaccines in Phase I, DNA vaccine encoding PfCSP and PfAMA-1 provided 27% sterile immunity [107]. Despite the challenges, DNA vaccines have been instrumental in establishing the feasibility of genetic immunization and in defining antigenic targets for next-generation vaccine designs.

mRNA vaccines have more recently emerged as a promising alternative, driven by advances in lipid nanoparticle (LNP) formulation and the successful deployment of mRNA vaccine technology against SARS-CoV-2 during the COVID-19 pandemic [108]. mRNA vaccines offer several advantages over conventional platforms, including precise antigen design via in silico methods and stimulate immune responses via both MHC class I and II, as well as have rapid scalability of production. Pre-clinical studies have demonstrated that LNP-encapsulated mRNA encoding PfCSP was immunogenic [109]; encoding Pfs25 and Pfs230D1 can elicit potent antibodies and maintain > 99% transmission, reducing activity in animal models [110]. Another study combining PfCSP and Pfs25 has shown that mRNA is an appropriate platform to aim for both pre-erythrocytic and transmission-blocking stages [111]. Early-stage investigations are exploring multivalent and self-amplifying mRNA constructs to improve immunogenic breadth and durability. One study focused on fusing PfCSP mRNA with a chemokine (MIP3α) reported improved immunogenicity [112].

Although there is a growing interest in mRNA technology, the platform faces several challenges. One major limitation is the intrinsic instability of mRNA, which often necessitates stringent cold-chain requirements that can complicate distribution in low-resource settings. Advances in formulation have improved stability profiles, storage, and transport, but infrastructure remains an important consideration for large-scale deployment in endemic regions. The efficient in vivo delivery relies on sophisticated lipid nanoparticle (LNP) systems, and while these have proven effective, optimization of uptake and dose efficiency continue to be areas of active research. Reactogenicity is another factor requiring careful monitoring as mRNA vaccines might trigger unintended immune overactivation, causing side effects like inflammation or rare myocarditis. Manufacturing complexity, reliance on specialized raw materials and facilities, and associated costs may limit the large-scale scalability of mRNA vaccines in low-income settings. In addition, the transient nature of mRNA-driven antigen expression may require booster dosing to maintain durable protection. Nevertheless, ongoing advances, such as improved LNP formulations, enhanced thermostability, and self-amplifying mRNA constructs, aim to increase stability, reduce dose requirements, and improve accessibility in malaria-endemic regions.

## 4. Vaccine Adjuvants That Have Been Used in Malaria Research

Vaccines must deliver a target antigen and provide immune-stimulating signals to induce an effective immune response [113]. While some vaccines, such as viral-vectored vaccines, naturally provide immune stimulation through their skeleton, others require additional immunostimulatory components called adjuvants [114]. Adjuvants are particularly important for protein and polysaccharide-based subunit vaccines, which, unassisted, have a weak immunogenic profile [115]. They enhance vaccine efficacy by shaping both the magnitude and quality of the immune response. Adjuvants promote B cell affinity maturation, broadening humoral immunity and improving the durability of antibody and memory T and B cell responses [114,115,116]. In addition, they influence the size and phenotype of memory cells and may imprint long-term effects on innate immunity, contributing to sustained protection against infection [117,118].

Adjuvants vary in the composition and mechanisms of action. They can be grouped as depot-forming adjuvants, emulsions, saponins, DAMP/PAMP agonists, and combination adjuvant formulations in which small molecules are incorporated into liposomes or nanoemulsions [119]. Early adjuvants were developed to enhance overall immunogenicity without targeting specific immune pathways; nonetheless, their biological effects are multifaceted [120]. In contrast, newer adjuvants are designed to engage defined molecular sensors, such as TLRs, which allow for more precise immune modulation [114]. Lipid nanoparticles (LNPs), the carriers for mRNA vaccines, exemplify this new generation of adjuvants as they not only deliver antigens but are also capable of activating innate immunity through mechanisms that remain to be determined [121]. Several adjuvants have been investigated in malaria vaccine development, each with distinct immunostimulatory properties that influence both response magnitude and quality. Aluminum-based adjuvants (alum) are among the most used due to their strong safety profile; they primarily induce a T helper 2 (Th2)-skewed response. Although alum was once thought to act mainly by forming antigen depots, later studies demonstrated that this is not necessarily the case for every single antigen [122,123]. Since alum also induces a local inflammation via cell death at the injection site, this facilitates antigen uptake and transport to dLNs [124]. AS01, a liposome-based adjuvant system containing 3-O-desacyl-4′- MPLA and QS-21, is used in the RTS,S vaccine and elicits both strong T cell-mediated and humoral immune responses [9,125]. AS02, an oil-in-water emulsion also containing MPLA and QS-21, was tested in earlier RTS,S formulations. Matrix-M, another saponin-based adjuvant, has demonstrated the ability to stimulate both humoral and cellular immune responses and is currently used in the R21 malaria vaccine [18]. It is composed of three primary components: saponins extracted from the bark of the *Quillaja* *saponaria* (soapbark) tree, cholesterol, and phospholipids. These are formulated into two nanoparticle types: Matrix-A^TM^, containing Fraction-A saponins with lower reactogenicity, and Matrix-C^TM^, enriched with Fraction-C saponins offering stronger adjuvant potency. The final Matrix-M^TM^ formulation combines these in an 85:15 ratio, yielding ~40 nm cage-like nanoparticles that enhance antigen delivery and immune stimulation [126]. Matrix-M induces durable antibody production, strong T cell activation, and efficient antigen drainage to local lymph nodes [127]. It also provides antigen dose-sparing effects and promotes a balanced Th1/Th2 CD4^+^ T cell response, thereby enhancing the breadth and functional diversity of IgG subclasses [128]. Beyond malaria, Matrix-M has been incorporated into licensed or late-stage vaccines for COVID-19 (Novavax) [129,130], seasonal influenza [131], and Ebola virus disease [132].

Other promising adjuvants include GLA-SE (Glucopyranosyl Lipid Adjuvant-Stable Emulsion), a TLR4 agonist that enhances Th1 cell-mediated responses and has shown potential in clinical trials for malaria vaccines [43]. CAF01, a liposomal adjuvant composed of cationic liposomes and a synthetic mycobacterial glycolipid, has been tested with the GMZ2, a chimeric blood-stage protein vaccine against GLURP and MSP3 [133]. Additionally, CpG oligodeoxynucleotides (CpG ODN), Montanide ISA 51 and 720, and GLA-LSQ have been explored in clinical trials for malaria vaccines [73,134,135,136]. More recently, 3M-052, a synthetic TLR-7/8 agonist, combined with aluminum hydroxide has demonstrated robust immune responses in non-human primate (NHP) studies with R21 [137]. Similarly, the combination of VLP vaccine candidates with Cquim-MA adjuvant, a dual TLR7/8 agonist, enhances the immune response, resulting in a more robust protection in malaria immunization [138,139].

## 5. Bottlenecks

The recently WHO-recommended malaria vaccines have been a major step-forward in combating malaria infections, with RTS,S and R21 both being used and recommended by the WHO. However, despite the success of these vaccines, limitations remain, including their limited capacity to induce sufficient cellular and tissue-resident immune responses (particularly CD8^+^ T cells) due to the mechanism of VLP-induced immunity [78], as well as the induction of minimally somatically hypermutated B cells [140].

**Figure 2:**
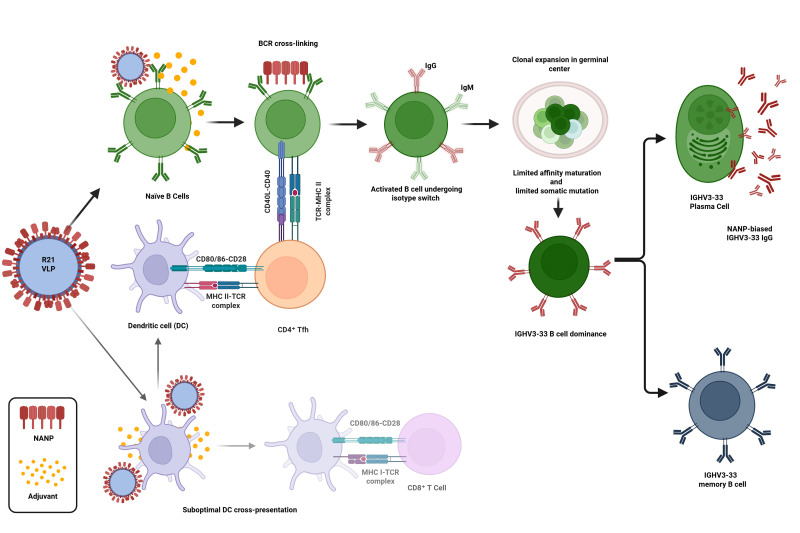
NANP-decorated VLP vaccine biases humoral immunity over cytotoxic cellular response. Following administration, VLPs are taken up by antigen-presenting cells, where they are processed and the NANP repeats are presented on major histocompatibility complex (MHC) class II molecules to CD4^+^ T cells. In parallel, the highly repetitive display of NANP epitopes on VLPs enables cross-linking of B cell receptors, which promotes B cell activation and germinal center formation. Within germinal centers, affinity maturation drives the differentiation of B cells into long-lived antibody-secreting plasma cells and memory B cells. In contrast, VLPs generally induce limited CD8^+^ T cell activation and are less effective at eliciting cytotoxic T cell response. Figure was created via BioRender.com.

Highly repetitive sequences, as found in the NANP region of CSP, can mediate strong B cell receptor (BCR) cross-linking [141] and induce both T-independent and T-dependent B cell maturation, leading to perturbed affinity maturation [142,143]. As studies go into more depth on the mechanisms of vaccine-induced protective immunity against malaria, certain recurring B cell populations involving the selection of IGHV3-33 phenotypes are shown to dominate the frequency of memory B cell pools [141]. This gene has been linked with near germline B cells targeting the immunodominant NANP motifs, as well as showing some cross-reactivity with NVDP and NPDP sequences in the CSP junction domain [141]. Moreover, a study by McDaniel et al. analyzed IgG responses to NANP post-R21 vaccination before and after CHMI challenge, which found that ~70% of circulating NANP-reactive IgG were derived from IGHV3-33 populations [140]. Furthermore, this IGHV bias entailed significantly lower somatic hypermutation (SHM) rates for NANP-reactive IGHV3-33 IgG compared to non-reactive IgG [140], as illustrated in Figure 2. One factor that may explain this clonal selection of IGHV3-33 phenotypes is that these naïve memory B cell precursors, reactive to the immunodominant NANP domain of CSP, outcompete rare B cells, which may have higher antigen affinities following SHM [144]. VLP-based vaccines using the bacteriophages MS2 and Qβ to display the CSP L9 epitope induced strong protective immunity against malaria infection when administered in combination [139].

Despite its critical functions and accessibility on the sporozoite surface, targeting CSP alone presents limitations. Hepatocyte invasion is mediated by multiple proteins—including TRAP, SPECT-1, SPECT-2, CelTOS, and other liver-stage antigens—providing redundant pathways that may allow some sporozoites to evade CSP-directed immunity [145]. Moreover, CSP is expressed only during the brief extracellular phase of sporozoite migration from the skin to the liver [146]. Once hepatocytes are invaded, surface CSP is cleaved or down-regulated and no longer maintained as a dominant surface antigen, which limits the natural boosting of CSP-specific antibodies and contributing to waning immunity after vaccination [146,147].

Another caveat to current VLP vaccination against malaria is through the induction of predominantly humoral immune responses and memory [42]. The size and organization of VLPs allow for efficient trafficking into dLNs to induce a strong B cell germinal center formation [42,82]. Although the RTS,S and R21 vaccines contain CSP C-terminal TSR (thrombospondin type-I repeat) domains, which are known T cell epitopes [94], antibody formation against NANP repeat regions predominate the immune response induced by these vaccines [42]. On the contrary, field trial studies administering monoclonal antibodies (mAb) targeting junctional epitopes, like CIS43LS and L9LS mAbs, demonstrate 70–80% protection in individuals from parasitemia [148,149]. As indicated by these studies, antibodies targeting the junctional domain include CIS43LS and L9LS and are known to be protective through the recognition of NPDP and NVDP motifs, while both the RTS,S and R21 vaccines lack these sequences and rely on cross-reactive antibodies to target these regions [150]. As such, research into the reasons for the poor antibody targeting/immunogenicity of junctional domains during its inclusion in pre-clinical vaccination studies [151] should warrant further investigation since mAb therapies, as found with CIS43LS and L9LS, are capable of inducing sterile protection in humans [152]. Therefore, this may pave an avenue for reverse-engineering antigens to be used as vaccine candidates.

Research investigating the responses in animal models, from rhesus monkeys to mice, to attenuated sporozoite vaccination have been pivotal in increasing our understanding of the correlates of protection against malaria. One such factor, important in recognizing infected hepatocytes, is the role of Th1 responses in targeting liver merozoite development [153]. These not only include CD8^+^ T cells but also CD4^+^ Th1, NK and NKT cells, which are the primary producers of IFN-γ, a cytokine toxic to developing merozoites, through the induction of iNOS and Nitric Oxide (NO), as well as hepatocytes [153,154,155]. Therefore, because of the complex lifecycle of malaria involving transient extracellular phases along with intracellular development cycles, a vaccine regimen eliciting cellular arms of the immune system in conjunction to humoral responses would be able to target the parasites even after evading antibody responses, which is something VLP vaccines elicit to a low extent [156].

Several CHMI and NHP studies have attempted to explore the possibility of concomitantly administering VLP with viral vectored vaccines to induce robust antibody and cellular responses through CD8^+^ and CD4^+^ Th1 cells [26]. In a clinical study by Rampling et al., participants were vaccinated with RTS,S and ChAd63 ME-TRAP, with the aim of eliciting distinct immune responses against different antigens [26]. Although the regimen was safe and immunogenic, demonstrating no significant adverse effects and achieving sterile protection in approximately 70–80% vaccinated participants, no correlation was observed in the involvement of TRAP-specific T cell counts or CSP and TRAP-specific IgG responses [26]. Given the possibility of immune interference when targeting multiple epitopes simultaneously, a study using rhesus macaques by Pichyangkul et al. reported the suppression of AMA1-specific IFN-γ and antibody responses during concomitant vaccination with MSP1 [157]. Interestingly, this suppression was reversed when RTS,S was included in the regimen. Furthermore, in another NHP study, vaccination with radiation-attenuated *P. knowlesi* sporozoites led to parasitemia following the experimental depletion of CD8^+^ T cells using monoclonal antibodies; protection was restored once CD8^+^ T cells repopulated [158].

## 6. Future Directions

Due to the complex lifecycle of *Plasmodium* parasites, generating highly protective vaccines will likely require the inclusion or targeting of multiple epitopes and antigens derived from different stages of the parasite development. Several studies using multi-epitope vaccine administration targeting different stages demonstrate increased efficacy through the inclusion of additional steps at which the parasite can be targeted [103,159]. The importance of mixed-stage vaccines is further illustrated in Table 1 through the ongoing trails testing the efficacy and safety of combining pre-erythrocytic candidates with blood or sexual-stage vaccines. Such combination approaches aim to provide both individual protection against clinical disease and community-level benefits by reducing parasite transmission. Integrating vaccines that prevent symptomatic malaria with transmission-blocking components may enhance the overall acceptance of these vaccines. Notably, sexual-stage vaccines do not clear parasites from the vaccinated host, as the induced antibodies act within the mosquito midgut to inhibit parasite development after blood feeding. However, immune interference between co-administered antigens remains a concern. Rational antigen selection, the optimization of dosing intervals, and the careful evaluation of immunodominance hierarchies will be critical to ensure balanced and effective immune responses.

Advances in structural biology and computational immunology have enabled increasingly precise protective epitope mapping. In silico approaches, including epitope prediction tools such as the Immune Epitope Database (IEDB), have facilitated the identification of recurring MHC class I-restricted epitopes; notably, approximately 42% of predicted CD8^+^ T cell-recognized epitopes have been mapped to CSP [160]. Similar strategies are now being applied to other malaria antigens, comparing epitope recognition patterns in malaria-naïve versus naturally exposed individuals to better define protective targets. This approach has contributed to the optimization of next-generation constructs such as the improved RH5.2 antigen derived from RH5.1 [32]. Structure-guided design has also informed antibody-based interventions. The development of CIS43 monoclonal antibodies targeting the junctional epitope of CSP represents a prominent example. A modified long-acting variant, CIS43-LS, engineered for enhanced stability and extended half-life, has progressed to Phase II clinical evaluation (NCT04329104) [161]. To this end, it should be mentioned that with the advances in computer processing and machine learning, leveraging in silico prediction and bioinformatics highlights the possibility to pinpoint sequences based on a wide range of characteristics, like antigenicity and solubility with high accuracy, for pre-clinical vaccine studies and synthesis [162,163]. Moreover, in silico design may be fundamental in tailoring the immune response to certain epitopes on an antigen using tailored vaccine boosters to shepherd and polish immunogen targeting, a similar design used to vaccinate against HIV [164]. Complementary technologies such as immune repertoire sequencing, predictive modeling of vaccine responses, and refinement of CHMI models are poised to accelerate translational progress.

Despite these computational advances, experimental systems remain indispensable. Animal models, particularly murine models, have been central to malaria vaccinology by enabling detailed dissection of immune mechanisms underlying infection and protection. Although AI-driven modeling and in silico prediction are increasingly powerful tools for candidate prioritization, they currently complement rather than replace in vivo studies [165].

As the field advances, greater attention should also be directed toward malaria in vulnerable populations, including pregnant women and immunocompromised individuals [166]. Studies in malaria-endemic countries have demonstrated the protective roles of CD4/8^+^ T cells in infants who developed from malaria exposure in the placenta [167], and vaccination with radiation-attenuated sporozoites in pregnant women has been shown protection for up to two years [168]. Expanding preclinical maternal vaccine research using both small animal models [169], as well as organoid or placental explant systems [170], may help reduce pregnancy-associated morbidity. Further, vaccine combinations with different parasite lifecycle stages or strains could improve vaccine potency by providing stage and strain-transcending immunity, respectively.

Finally, given the coexistence of multiple malaria species in endemic regions and the recent successes of R21 and RTS,S vaccination, an important aspect that warrants investigation is the feasibility of combinatorial vaccination against *P. falciparum* and *P. vivax*. The ability of P. vivax to form dormant hypnozoites allows recurrent infections to occur even after clearance of the primary infection [171], presenting a major challenge for malaria control and elimination. Therefore, next-generation vaccine strategies should aim to induce high antibody titers capable of neutralizing parasites prior to hepatocyte invasion or elicit robust CD8^+^ T cell responses that target infected hepatocytes. Integrating these immune mechanisms into multivalent or multi-species vaccine platforms may help prevent relapse and provide broader protection. Such approaches will be critical for advancing toward long-term malaria control and eventual global elimination.

## Figures and Tables

**Table 1:** Ongoing clinical trials for malaria vaccine development. ChAd63, chimpanzee adenovirus 63; CSP, circumsporozoite protein; CyRPA, cysteine-rich protective antigen; GLA-SE, glucopyranosyl lipid A in stable emulsion; IM, intramuscular; IMX313, oligomerization domain derived from complement inhibitor C4-binding protein; IV, intravenous; LARC, late-arresting replication-competent; MVA, modified vaccinia Ankara; ME-TRAP, multiple epitope–thrombospondin-related adhesion protein; mRNA, messenger ribonucleic acid; MSP1, merozoite surface protein 1; PE, pre-erythrocytic; *Pf*, *Plasmodium falciparum*; Pfs25, sexual stage antigen 25; Pfs45/48, 45/48 kDa sexual stage antigens; RH5, reticulocyte-binding protein homologue 5; RIPR, RH5 interacting protein; SPZ, sporozoite; VLP, virus-like particle.

*Product Name*	*Antigen*	*Vaccine Platform*	*Route*	*Trial Phase*	*Clinical Trial ID*
**Pre-erythrocytic (PE) stage**					
BNT165e	PfCSP	mRNA	IM	Phase I/II	NCT06069544
PfSPZ-LARC2	Whole SPZ	Genetically attenuated SPZ	IV	Phase I	NCT06735209
PfSPZ; MVA/ChAd63 ME-TRAP	Whole SPZ, ME-TRAP	Genetically attenuated SPZ, viral vector	Mixed (IV, IM)	Phase I/IIa	NCT05441410
RTS,S/AS01E	PfCSP	VLP	IM	Phase IIa	NCT07036159
				Phase IV	NCT06424002
R21/Matrix M	PfCSP	VLP	IM	Phase II	NCT06879327
				Phase II	NCT06080243
				Phase II	NCT07074665
				Phase III	NCT06578572
				Phase IV	NCT06068530
				Phase IV	NCT06860178
				Phase IV	NCT07194668
**Blood Stage**					
MSP1/GLA-SE	PfMSP1	Protein subunit	IM	Phase I	NCT06652737
				Phase I	NCT06862453
RH5.1/Matrix M	PfRH5	Protein subunit	IM	Phase I	NCT06141057
RH5.1/Matrix M; R78C/Matrix M; RH5.1 + R78C/Matrix M	PfRH5, PfCyRPA, PfRIPR	Protein subunit	IM	Phase Ia	NCT05385471
RH5.1/Matrix M1; RH5.2-VLP/Matrix M1	PfRH5	Protein subunit and VLP	IM	Phase I/IIb	NCT05790889
RH5.1/Matrix M+ RH5.2-VLP/Matrix M; RH5.1/Matrix M; RhH.2-VLP/Matrix M	PfRH5	Protein subunit and VLP	IM	Phase I/IIa	NCT05978037
**Sexual stage**					
Pfs25-IMX313/Matrix M; Pfs45/48/Matrix M; Pfs25-IMX313+ Pfs45/48/Matrix M	Pfs25, Pf45/48	Protein nanoparticle	IM	Phase Ib/IIa	NCT06549257
**Multi-stage (Blood and PE)**					
R78C/Matrix M; RH5 + R78C/Matrix M + R21/Matrix M	PfCyRPA, PfRIPR; PfRH5, PfCSP	Protein subunit; VLP	IM	Phase IIb	NCT07183371
RH5.2-VLP/Matrix M1+ R21/Matrix M1; RH5.2-VLP/Matrix M1; R21/Matrix M1	PfRH5, PfCSP	VLP	IM	Phase Ib	NCT05357560
RH5 + R21 + R78C/Matrix M; RH5 + R21/Matrix M; R21 + R78C/Matrix M; RH5 + R78C/Matrix M; R21/Matrix M	PfRH5, PfCyRPA, PfRIPR; PfCSP	Protein subunit and VLP	IM	Phase Ib	NCT06958198

## Data Availability

No new data were created for this work; not applicable.
